# First person – Michael Robichaux

**DOI:** 10.1242/dmm.049558

**Published:** 2022-05-19

## Abstract

First Person is a series of interviews with the first authors of a selection of papers published in Disease Models & Mechanisms, helping early-career researchers promote themselves alongside their papers. Michael Robichaux is first author on ‘ Subcellular localization of mutant P23H rhodopsin in an RFP fusion knock-in mouse model of retinitis pigmentosa’, published in DMM. Michael conducted the research described in this article while a postdoctoral fellow in Ted Wensel's lab at Baylor College of Medicine, Houston, TX, USA. He is now an assistant professor at West Virginia University, Morgantown, WV, USA, investigating the intricate subcellular processes in photoreceptor neurons that enable vision.


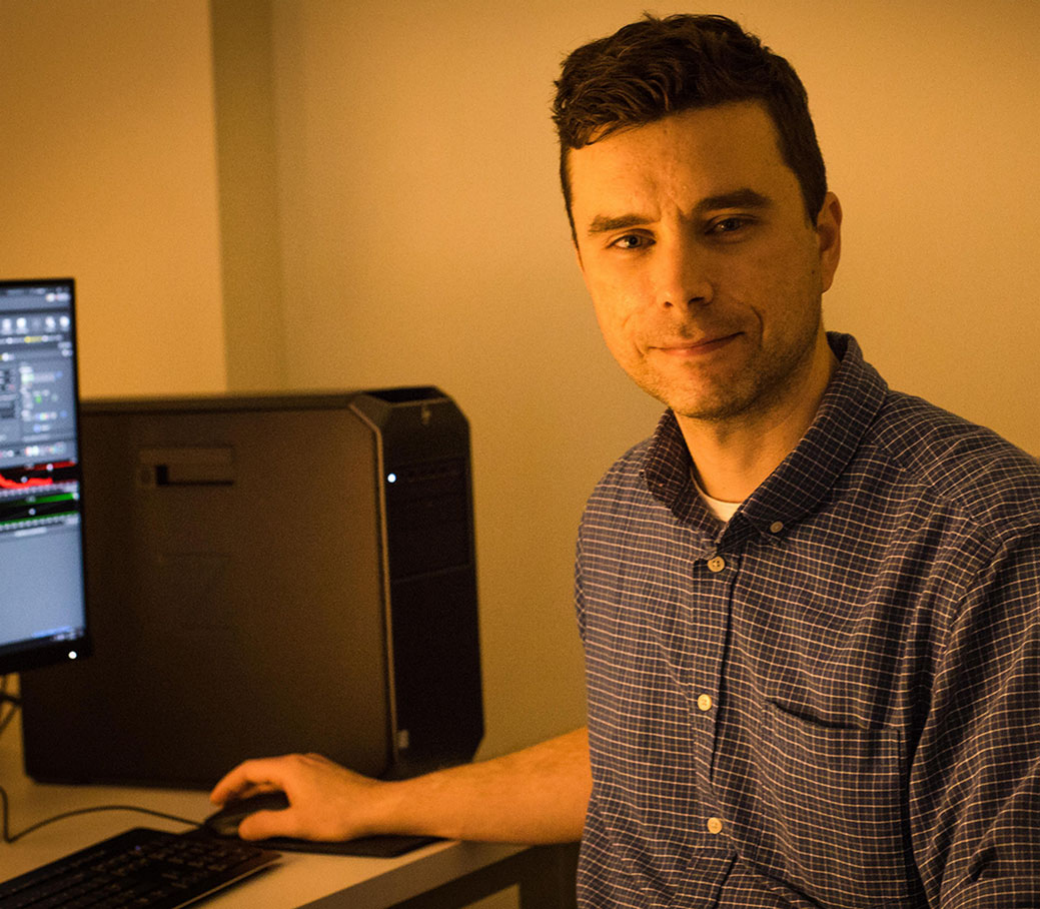


**Michael Robichaux**

**How would you explain the main findings of your paper to non-scientific family and friends?**

The pathology of diseases that cause gradual loss of eyesight and blindness are often rooted in the molecular workings of the cells in the retina of the eye. In the retina, photoreceptors sense light. Retinitis pigmentosa is a progressive blinding disease that is caused by proteins accumulating in photoreceptor cells in a harmful way, which leads to the death of these cells and light sensitivity in the retina to be impaired. In this paper, we generated a transgenic mouse model of retinitis pigmentosa that allowed our group to use advanced microscopy techniques to analyse this detrimental protein accumulation in a highly precise manner. The findings aid in our understanding of the molecular causes of blinding diseases and how they may be treated in the future.

“Our mouse model of retinitis pigmentosa is unique in that it introduces a bright fluorescent Tag-RFP-T protein tag onto the mutant P23H form of rhodopsin.”

**What are the potential implications of these results for your field of research?**

Our mouse model of retinitis pigmentosa is unique in that it introduces a bright fluorescent Tag-RFP-T protein tag onto the mutant P23H form of rhodopsin. With this tag, the aggregation of mutant rhodopsin in rod photoreceptors can be traced over time in longitudinal gene therapy studies, for example.

**What are the main advantages and drawbacks of the model system you have used as it relates to the disease you are investigating?**

The advantage of our model is that the aggregation of the Tag-RFP-T-tagged mutant P23H-Rhodopsin protein in the endoplasmic reticulum of rod photoreceptors is very apparent and easy to detect from mouse retinal samples prepared for either fluorescence or electron microscopy. A drawback to the model is that we cannot distinguish the role that the Tag-RFP-T fluorescent protein tag plays on the misfolding and aggregation properties of the mutant rhodopsin. Another limitation is that we were unable to identify the cause of the mild retinal degeneration observed in this mouse model, which remains a challenge for us.

**Figure:**
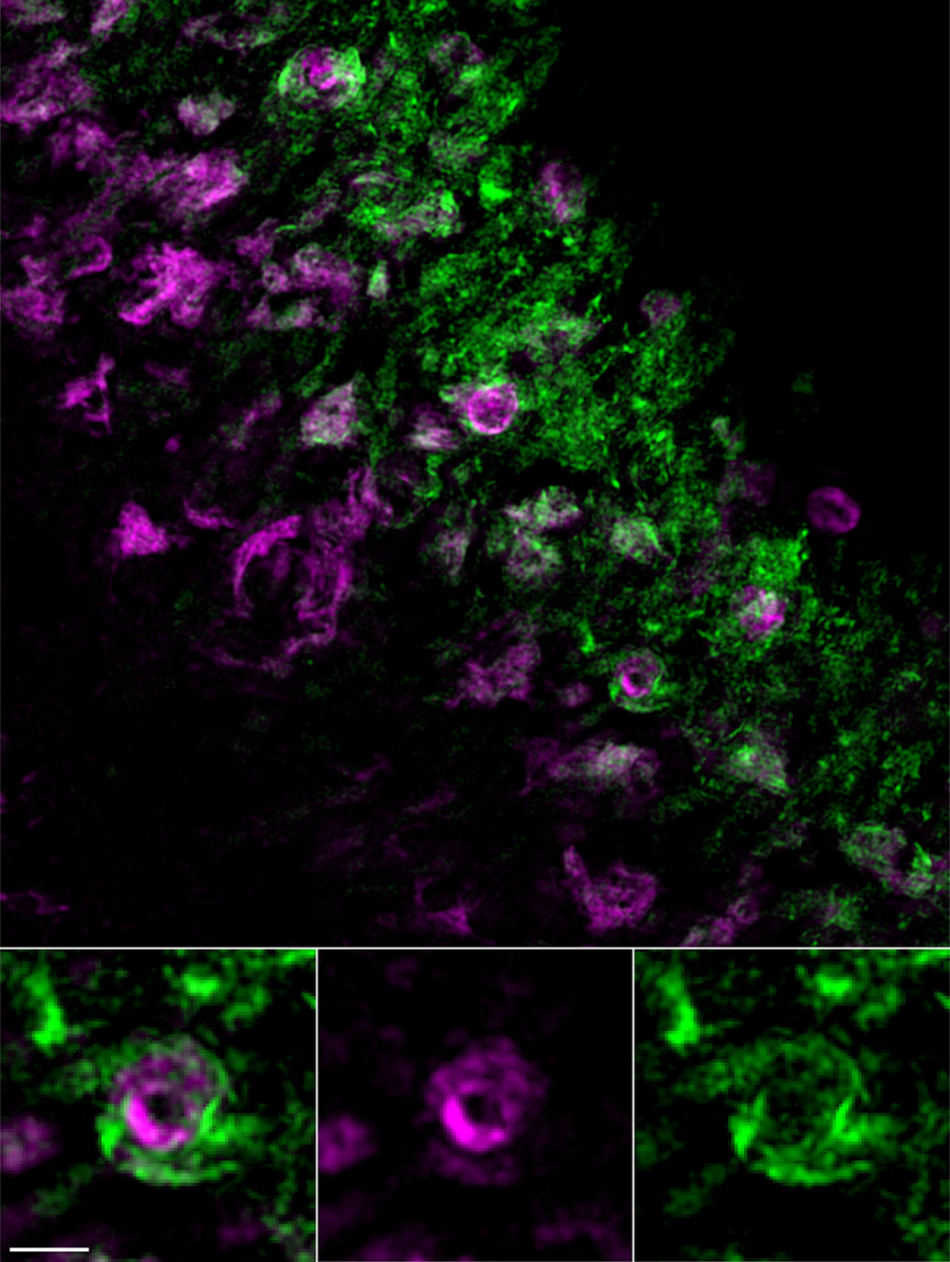
**The GFP/RFP hurricane! In the cytoplasm of a rod photoreceptor neuron in a P23H-rhodopsin-Tag-RFP-T heterozygous mouse retina, wild-type rhodopsin tagged with eGFP (green) is wrapped in a vortex around retinitis pigmentosa mutant P23H-Rho protein tagged with Tag-RFP-T.** Images were acquired with structured illumination microscopy. Scale bar: 1 µm.

**Describe what you think is the most significant challenge impacting your research at this time and how will this be addressed over the next 10 years?**

There is a strong motivation to develop therapies for eye diseases, but I believe it is still a challenge for the field to pursue the difficult unanswered basic science questions about how photoreceptor neurons work. I am optimistic that the development of new and exciting tools and techniques will help us take on the challenge!

“A full embrace of open science and the free exchange of ideas would greatly assist early-career scientists in doing the work they are passionate about.”

**What changes do you think could improve the professional lives of early-career scientists?**

I think a full embrace of open science and the free exchange of ideas would greatly assist early-career scientists in doing the work they are passionate about.

**What's next for you?**

The saga continues in my new lab at West Virginia University, where my small but valiant team is already producing amazing results. Shout out to Kristen, Shannon and Samantha!
